# 肺癌合并肺栓塞：抗凝评估和管理的研究进展

**DOI:** 10.3779/j.issn.1009-3419.2025.101.25

**Published:** 2025-12-20

**Authors:** Yiming MA, Kun LIU, Changling TU, Tongyao XU, Bolin LIU

**Affiliations:** ^1^650118 昆明，昆明医科大学第三附属医院老年肿瘤科; ^1^Department of Geriatric Oncology, The Third Affiliated Hospital of Kunming Medical University, Yunnan Cancer Hospital, Peking University Cancer Hospital Yunnan, Kunming 650118, China; ^2^650500 昆明，昆明医科大学; ^2^Kunming Medical University, Kunming 650500, China

**Keywords:** 肺肿瘤, 肺栓塞, 抗凝治疗, 风险评估, Lung neoplasms, Pulmonary embolism, Anticoagulants, Risk assessment

## Abstract

肺癌是全球发病率和死亡率最高的恶性肿瘤，肺癌患者发生静脉血栓栓塞症（venous thromboembolism, VTE）的风险高，尤其肺栓塞（pulmonary embolism, PE），不仅会延长住院时间、增加住院费用，还严重影响患者的生存期和生活质量。目前关于肺癌患者发生PE的风险评估模型及出血风险评估模型效能有限，临床医生在选择抗凝或预防出血方面面临挑战。此外，抗凝过程中还面临着药物相互作用及特殊人群管理等复杂问题。本文通过回顾既往文献，总结抗凝与出血的临床风险评估和管理、抗凝药物的选择及肺癌合并PE患者的二级预防，为临床个体化诊断和治疗提供参考。

## Abstract (en)

Lung cancer is the malignant tumour with the highest incidence and mortality rates globally. Patients with lung cancer face a high risk of developing venous thromboembolism (VTE), particularly pulmonary embolism (PE). This not only prolongs hospital stays and increases inpatient costs but also severely impacts patient survival and quality of life. Current risk assessment models for PE and bleeding risk in lung cancer patients remain limited in efficacy, posing challenges for clinicians in selecting anticoagulation or preventing bleeding. Additionally, anticoagulation management involves complex issues such as drug interactions and managing special populations. Based on existing literature, this review summarizes the clinical risk assessment and management of anticoagulation and bleeding, the selection of anticoagulants, and secondary prevention strategies for lung cancer patients with PE, to provide a reference for individualized clinical diagnosis and treatment.

肺癌是全球发病率和死亡率最高的恶性肿瘤，静脉血栓栓塞症（venous thromboembolism, VTE）是全球第三大常见死因，VTE包含肺栓塞（pulmonary embolism, PE）和深静脉血栓^[1]^。术前VTE调查显示，肺癌患者患VTE的概率达9.6%^[2]^，肺癌的组织学类型和分子亚型都会影响VTE的发生^[3]^；一些治疗方式，比如使用表皮生长因子受体-酪氨酸激酶抑制剂（epidermal growth factor receptor-tyrosine kinase inhibitors, EGFR-TKIs）、免疫检查点抑制剂（immune checkpoint inhibitors, ICIs）及手术也会影响VTE的发生风险^[4-6]^。因此加强肺癌合并PE发生风险的评估，注重个体化的诊断和治疗是临床工作中需要重视的部分。建立全程化管理模式，有利于改善肺癌患者的预后。

癌症患者本身存在促凝血因子分泌的改变，进而导致促凝和抗凝的不平衡^[7]^。肺癌患者属于出血的高风险人群[风险比（hazard ratio, HR）=2.1]^[8]^，肺癌的治疗因素，如使用抗血管生成药物可能引起出血风险增加^[9]^。由于肺癌患者出凝血机制的复杂性和药物相互作用等特殊因素，在抗凝的同时注重患者的出血风险，进行精准评估并选择合适的治疗方式，对于确保治疗的有效性和安全性十分重要。

## 1 VTE评估工具的运用现状和展望

### 1.1 VTE评估工具的现状

在临床治疗中，通常先进行VTE风险评估，再对PE做预测。PE作为VTE最危重的表现形式之一，绝大多数源于下肢深静脉血栓脱落，因此，若能发现早期的血栓问题，并进行干预，将大大减少PE这一急症。针对肺癌合并VTE的预测模型有待探索。目前用于肿瘤患者抗凝风险评估的量表是“Khorana评分”，然而一项大规模的真实世界研究^[10]^表明，该评分在肺癌合并VTE的高风险组患者中，6个月VTE的绝对风险为4.1%。一篇荟萃分析^[11]^显示：Khorana评分在不同癌种（包括肺癌）中的C-index均低于0.7。Khorana评分在肺癌患者中预测能力有限，肺癌患者预测VTE需要全新的、针对性强的预测手段。

### 1.2 VTE新兴评估工具的表现

近年来针对肺癌患者的VTE风险评估涌现出了一些新的工具，包括COMPASS-CAT、改良Caprini、CATS/CONKO、Thrombo-NSCLC。一项荟萃分析^[12]^显示，肺癌患者中，现有的Khorana、PROTECHT、CONKO评分的区分能力有限，相比之下COMPASS-CAT评分与高风险VTE存在显著关联[相对危险度（relative risk, RR）=4.68]，适用于高风险VTE的筛查。另一项回顾性队列研究^[13]^表明，COMPASS-CAT评分在识别接受化疗或ICIs的非小细胞肺癌（non-small cell lung cancer, NSCLC）这类VTE高危人群方面的表现显著优于Khorana评分。一项回顾性队列研究^[14]^表明针对接受ICIs治疗的患者，Khorana评分的预测效能不足[曲线下面积（area under the curve, AUC）=0.553]，而Caprini和Padua评分则仍然保持着较好的预测效能（AUC分别为0.743和0.745），这提示面对使用ICIs治疗的肺癌患者，需要合理地选择评估工具。一项多中心观察性的队列研究^[15]^在原有Caprini风险评估模型中整合了D-二聚体和新的手术时间分层，使原有的评估模型预测能力提高（AUC: 0.589 *vs* 0.759），适用于肺癌术后患者的VTE风险分层。针对门诊化疗的肺癌患者，现有的模型主要有Khorana、PROTECHT、CONKO、CATS和COMPASS-CAT评分。现有的评分模型中，有一些基于Khorana评分为肺癌合并VTE患者做了改良，一项前瞻性队列研究^[16]^对这几项评分进行比较，只有CONKO、CATS评分对VTE的发生进行分层，CATS和CONKO评分在Khorana评分的基础上整合了化疗方案以及性别、年龄等，并基于D-二聚体和美国东部肿瘤协作组（Eastern Cooperative Oncology Group, ECOG）体能状态构建了新模型，提升门诊肺癌患者的VTE预测精度与风险分层能力。一项单中心前瞻性队列研究^[17]^通过加入VTE、可溶性P-选择素和凝血因子VIII构建了针对门诊NSCLC患者的Thrombo-NSCLC评分，其预测效能远优于Khorana评分（AUC: 0.93 *vs* 0.55），然而该研究的样本量较小，且为单中心研究，其准确性有赖于外部验证。

### 1.3 治疗因素与VTE发生的关系

一项系统评价与荟萃分析^[18]^表明使用ICIs治疗的晚期NSCLC患者中，ICIs治疗相关的VTE发生率为1.9%。一项探讨程序性细胞死亡配体-1（programmed cell death ligand 1, PD-L1）是否会影响VTE发生和出血的单中心回顾性研究^[19]^表明，使用ICIs治疗的患者VTE发生率增加，但与PD-L1表达并无统计学关联。与使用第一、二代的EGFR-TKIs的患者相比，使用第三代EGFR-TKIs的肺癌患者发生VTE风险更高（HR=1.26），且VTE发生概率与临床亚型相关^[4]^。大型的癌症手术后，肺癌的VTE发生率与膀胱癌并列第一（发生VTE风险超过其他癌症2.5%）^[5]^。

### 1.4 新兴生物标志物对肺癌患者VTE的预测潜力

增加影响VTE的新的生物标志物可以完善Khorana量表，提升其对肺癌患者VTE的预测效能。间变性淋巴瘤激酶（anaplastic lymphoma kinase, *ALK*）重排的NSCLC患者的VTE发生率相较于*EGFR*突变型和野生型的患者高（*ALK*突变、*EGFR*突变和野生型患者的VTE发生率分别为43.5%、21.2%和17.2%）^[3]^。一项回顾性队列研究^[20]^也显示，*ALK*阳性患者VTE的发生风险高出*ALK*野生型的患者4倍（HR=3.70, 95%CI: 2.51-5.44），且*ALK*阳性复发VTE的概率也很高[比值比（odds ratio, OR）=4.85]。一项针对NSCLC和黑色素瘤患者荟萃分析^[18]^表明，联合用药对比单药ICIs会增加VTE发生率（3.1% *vs* 1.1%），这表明使用ICIs，尤其是联合治疗可能与VTE风险有关，但需更多前瞻性研究验证。一项多队列诊断研究^[21]^中，循环肿瘤DNA（circulating tumor DNA, ctDNA）阳性是VTE发生的独立风险影响因素，基于ctDNA的预测模型效能显著优于Khorana评分（C-index: 0.54-0.61）。另一项开放标签的队列研究TARGET-TP试验^[22]^通过依据生物标志物（纤维蛋白原和D-二聚体）对肺癌和胃肠道癌患者进行风险分层再进行抗凝，高风险组发生VTE的风险是低风险组的3.33倍。此外还有上文提到的D-二聚体升高（HR=1.124）^[16]^、可溶性P-选择素（HR=66.40）、凝血因子VIII升高（HR=4.15）^[17]^在NSCLC中有极强的预测能力。一项针对Caravaggio研究数据的回顾性研究^[23]^提示，VTE复发的独立预测因素包括深静脉血栓、ECOG体能状态评分≥1分、胰腺或肝胆管癌、接受抗癌治疗、高肌酐清除率。

### 1.5 机器学习模型的未来发展趋势

在使用机器模型预测肺癌合并VTE方面，一项前瞻性队列研究^[24]^基于XGBoost算法开发和验证了一个机器学习模型，通过引入新型生物标志物vWF-A2与临床病理变量构建了综合预测模型，在预测电视辅助胸腔镜手术（video-assisted thoracoscopic surgery, VATS）肺段切除早期的NSCLC患者中，预测效能显著优于传统的Caprini评分（AUC=0.856）。

### 1.6 PE严重程度与预后的评估工具

临床工作中，常用肺栓塞严重指数（pulmonary embolism severity index, PESI）来评估PE的严重程度，判断发生PE的患者适合住院治疗、家庭治疗还是门诊随访。一项回顾性队列研究^[25]^证明PESI通过风险分级能够预测PE患者30 d到5年的死亡率。一项回顾性开发与内部验证研究^[26]^开发了MAUPE-C，该模型有助于识别30 d内低风险的癌症合并PE患者，然而目前该量表仅经过内部验证，在癌症患者中区分30 d死亡风险方面优于RIETE和sPESI（simplified PESI）评分（AUC: 0.77 *vs* 0.64 *vs* 0.57），有待外部验证。一项针对确诊为PE患者的前瞻性、观察性单中心队列研究^[27]^发现，糖类抗原125（carbohydrate antigen 125, CA125）有较强的预测PE患者短期死亡率的能力，是预测PE患者30 d死亡率的独立影响因素（调整OR=4.95）。2023年的一项荟萃分析^[28]^针对疑似PE的患者开发了PE个体化预测模型，该模型包含患者年龄、是否患癌症、D-二聚体等变量，显著优于传统的Wells评分联合D-二聚体方案，然而，该模型并非针对肺癌。sPESI评分适用于临床快速评估PE风险，而PESI在长期预后的判断上更具优势^[25]^。

## 2 出血风险的评估现状和展望

### 2.1 癌症相关VTE患者出血风险评估工具的现状

目前癌症相关VTE患者的出血风险评估工具预测能力有限，一项观察性的队列研究针对7个量表（ACCP-VTE、HAS-BLED、Hokusai、Kuijer、Martinez、RIETE、VTE-BLEED）对癌症相关VTE患者出血风险评估的研究^[29]^表明，所有评分的预测性能都比较普通（C-index: 0.50-0.57），CAT-BLEED的表现略优（C-index: 0.63）。针对VTE合并癌症的住院老年患者，目前评分预测效能也相对局限，RIETE的C-index高，为0.61^[30]^。一项回顾性单中心模型研究^[31]^针对使用低分子肝素（low molecular weight heparin, LMWH）的出血风险构建了预测模型，纳入HAS-BLED出血风险评分、LMWH剂量和血小板计数几个因素，该模型可以简便地评估用药后的出血风险，帮助医生个体化地为患者制定LMWH的使用方案。针对目前癌症患者的出血风险评估量表和对应研究^[29]^，我们进行了横向对比，RIETE评分适用于65岁以上患者，但对大多数癌症合并VTE患者不适用^[30]^；CAT-BLEED评分仅适用于抗凝治疗的前6个月，且尚需外部验证。

### 2.2 肺癌合并VTE出血发生的特殊因素

一项2022年的VTE管理国际专家共识^[32]^指出，如果抗血管治疗发生VTE，要谨慎考虑是否引入抗血管生成药物进行抗肿瘤治疗。一项回顾性研究^[9]^发现，所有的抗血管生成药物（如贝伐珠单抗、阿柏西普、安罗替尼、舒尼替尼等）都会增加肺癌患者VTE和出血的发生风险。一项单中心、非干预性、回顾性队列研究^[8]^发现癌症相关VTE患者的出血率与转移癌发生部位有关，其中肺转移患者属于高风险类型（RR=2.0, 95%CI: 1.3-3.1），肺转移被确定为出血的长期独立预测因素（HR=2.1, 95%CI: 1.3-3.6），这提示在平衡出血风险和抗凝时需要考虑癌症转移部位。

### 2.3 机器学习模型在出血风险预测中的进展

一项机器学习模型研究^[33]^纳入了临床数据（患者基本信息、病史、治疗情况）、生物化学数据（各种血液指标，如血红蛋白、血小板计数、肾功能指标等）和患者的合并症及疾病谱，通过机器学习模型（如*LASSO*回归、XGBoost），针对癌症相关VTE患者1-90 d出血风险的预测，预测性能优于传统的CAT-BLEED评分（AUC: 0.48 *vs* 0.64）。我们认为，机器学习模型在性能上优于传统的评分原因主要是机器学习模型纳入的属性多，相较于传统的评分更接近真实世界数据；能纳入一些难以进行量化的非线性因素；还能够学习因素之间的交互作用。正因如此，机器学习模型具有针对性强的特点，更适合用于癌症患者的出血风险预测。然而，机器模型在临床运用中效能有限，可能原因是训练集中的出血患者较少，模型倾向于将样本预测为不出血来优化整体准确率，如何提升机器模型在临床中的准确度依然是研究难点。

## 3 肺癌合并PE抗凝药物的研究进展

### 3.1 LMWH应用的相关研究

肺癌合并PE的治疗，需基于不同危险分层采取相应处理措施。急性期需要着重溶栓治疗，慢性期则需做好长期抗凝。常用的抗凝药物主要有传统LMWH和因子Xa抑制剂等直接口服抗凝药物（direct oral anticoagulants, DOACs)。一项大型随机对照试验FRAGMATIC试验^[34]^表明，对未经选择的肺癌患者使用预防剂量的LMWH抗凝虽然降低了VTE风险，但增加了临床相关的出血风险。

### 3.2 DOACs的疗效研究

2023年美国临床肿瘤学会（American Society of Clinical Oncology, ASCO）指南^[35]^将阿哌沙班新增为VTE治疗的推荐选项（ASCO指南强推荐）。一项随机非劣效性对照试验^[36]^证明，比较利伐沙班和LMWH对预防肺癌术后VTE的发生率，利伐沙班的疗效和安全性不劣于LMWH，但其出血风险高于LMWH组（12.2% *vs* 7.0%, *P*=0.08），尤其是胃肠道出血风险。一项真实世界研究^[37]^表明，在针对癌症相关的VTE延长治疗中，相较LMWH，使用DOACs阿哌沙班可以降低VTE复发风险（HR=0.42），且具有更低的出血风险，这为DOACs作为更多无特殊并发症癌症患者长期抗凝的选择提供了支持。一项针对中国肺癌合并PE患者的真实世界研究^[38]^证明，利伐沙班的疗效不劣于LMWH。一项多中心的前瞻性队列研究^[39]^也表明，对于癌症合并症状性VTE患者，LMWH长期治疗存在依从性和耐受性差的问题，因此在肺癌患者中应该优先考虑DOACs。但在DOACs的选择上，要关注药物间的相互作用，一项动物试验^[40]^表明，当阿美替尼与利伐沙班联用，会增加利伐沙班的血药浓度，这提示若二者在临床联合使用，要严密监测并考虑剂量调整。

### 3.3 抗凝药物的选择

2024年美国国立综合癌症网络（National Comprehensive Cancer Network, NCCN）指南强调将DOACs作为无禁忌证患者的首选抗凝方案，临床应该结合患者的胃肠道功能、肾功能以及是否存在药物相互作用来选择DOACs^[41]^。一篇针对癌症合并VTE管理专家共识^[32]^系统梳理了一份决策框架，该框架涉及的核心关注点主要包括：是否针对特定人群抗凝、抗凝药物和抗癌药物是否联合使用、抗凝时长、针对血小板减少需如何管理以及肾功能不全时怎样做抗凝药剂量调整等，为临床医生提供了一个可行的决策流程。对于特殊人群抗凝药物的选择，一项观察性队列研究^[42]^表明，肾功能不全患者中，重度肾功能不全的癌症患者，致死性PE比致死性出血发生率高（2.3% *vs* 0.8%），因此对于肾功能不全患者需考虑抗凝。Caravaggio试验^[43]^表明，阿哌沙班治疗中度肾功能不全的患者[肌酐清除率（creatinine clearance, CrCl）：30-59 mL/min]比达肝素（一种低分子肝素）可能更有优势。对于轻度肾功能不全的患者（CrCl≥60 mL/min），建议选择DOACs，通常无需调整抗凝剂量；对于中度肾功能不全患者（CrCl: 30-59 mL/min），应优先选择阿哌沙班治疗，并调整剂量；对于重度肾功能不全和终末期肾病患者（CrCl<30 mL/min），应使用LMWH；对于终末期肾病患者（CrCl<15 mL/min），应暂停抗凝^[32]^。对于肝功能不全、血小板减少、体重处于极端范围的人群，抗凝策略尚无随机对照试验（randomized controlled trial, RCT）证据支持，专家共识推荐依据指南进行用药^[44,45]^。对于脑转移及颅内病变患者，一项基于数据库的回顾性队列研究^[46]^表明，颅内出血风险更多与癌症类型相关。一项回顾性队列研究^[47]^表明，肺癌手术患者围术期进行LMWH抗凝后，手术安全性无统计学差异。对于肝功能不全患者，需要依据Child-Pugh分级进行DOACs和LMWH的选择，Child-Pugh A级患者抗凝推荐阿哌沙班、艾多沙班、利伐沙班，且无需减量；Child-Pugh B级禁用利伐沙班，选用阿哌沙班、艾多沙班时需谨慎；Child-Pugh C级禁用所有DOACs^[44]^。对于极端肥胖患者，推荐使用LMWH抗凝，并检测凝血因子Xa保证疗效安全，使用DOACs需格外谨慎，必要时进行血药浓度监测^[45]^。一项动物实验^[40]^显示，阿美替尼使利伐沙班最大血药浓度和药时AUC增加，二者同时使用会增加出血风险。有研究^[9,32]^表明，所有的血管内皮生长因子（vascular endothelial growth factor, VEGF）或VEGF受体（VEGF receptor, VEGFR）的药物都会增加出血风险，其中VTE风险仅与VEGF/VEGFR靶向生物制剂有关[相对OR（relative OR, ROR）：3.11]，国际专家共识建议使用抗血管生成药物需要充分评估。一项药代动力学模型研究^[48]^表明，利伐沙班与厄洛替尼合用时会影响利伐沙班的代谢。

### 3.4 肺癌合并PE的溶栓治疗

一项单中心回顾性研究^[49]^表明，患有活动性肿瘤的患者采用溶栓治疗是影响PE患者1年内死亡的独立预后因素，HR高达8.07（95%CI: 2.72-23.98），这表明对于癌症患者这一高死亡风险（HR=3.04）的群体，须谨慎考虑是否在发生PE时采取溶栓。在癌症合并高危PE的患者中，肺癌亚组的死亡率很高（调整OR=2.68）^[46]^。一项回顾性观察性队列研究^[50]^表明，癌症患者接受溶栓后出血风险并未显著增加，而癌症患者的溶栓治疗却比非癌症患者更加保守。另一项大规模回顾性横断面研究^[51]^显示，肺癌合并PE患者使用溶栓治疗的出血性卒中发生率高（调整OR=1.75），临床医生面临是否溶栓的决策困境，需要更多的出血评估量表对该类型患者进行充分评估，以决定是否进行溶栓。

## 4 抗凝时间的抉择与二级预防

除了抗凝药物的选择，抗凝疗程的制定也至关重要，指南推荐活动癌症患者应该延长抗凝治疗时间^[35]^。一项回顾性单中心研究^[52]^采用竞争性风险分析表明，癌症相关VTE患者在延长抗凝的过程中，出血风险随着延长的时间呈线性下降，这表明当患者处于癌症活动期，延长抗凝有利于减少出血风险。ONCE PE试验^[53]^显示，对于癌症活动期合并PE的患者将利伐沙班的治疗时间从6个月延长到18个月能使PE复发的风险降低，6个月治疗组的复发率为19.1%，18个月治疗组的复发率为5.6%，发生大出血的风险有所增加（7.8% *vs* 5.6%）但无统计学意义。随机双盲试验EVE试验^[54]^的结果表明：在完成6-12个月初始治疗后，使用不同剂量的阿哌沙班继续进行二级预防，VTE发生率无明显差异，而在出血风险中也未显示统计学优势（8.9% *vs* 12.2%, HR=0.72, 95%CI: 0.38-1.37, *P*=0.39）。与之相比，一项高质量随机对照试验API-CAT试验^[55]^则表明，对于已完成6个月抗凝的VTE患者，在随后的12个月中，采用减量阿哌沙班（2.5 mg, *bid*）和全剂量阿哌沙班（5 mg, *bid*）进行抗凝，减量阿哌沙班的疗效不劣于全量阿哌沙班，而减量阿哌沙班安全性显著优于全剂量阿哌沙班，能够显著降低临床相关的出血事件（12.1% *vs* 15.6%, HR=0.75, 95%CI: 0.58-0.97, *P*=0.03）。基于该实验，针对需要进行二级预防的肺癌合并PE患者，应该考虑减量抗凝。两项研究都表明减量抗凝有益于长期维持治疗，但在出血风险的结论上有出入，出现这个结果的原因我们考虑有3点：（1）EVE试验是一项小样本量试验，而API-CAT试验属于一项大样本试验，API-CAT的试验结果更加稳定；（2）EVE试验是一项优效性试验，其研究终点是证明减量抗凝方案相较于全剂量抗凝能否减少出血风险，而API-CAT试验是一项非劣效性试验，其终点则是证明减量抗凝在预防VTE方面不劣于全量抗凝，研究终点不同，造成了结论强度的差异；（3）从基线特征上看，EVE试验的肺癌患者比例与API-CAT试验的肺癌患者比例接近，但比例都相对较低，因此在将结论外推至肺癌患者时还需谨慎。两组中API-CAT试验的PE患者比例更高（2.5 mg组：59.2%；5 mg组：56.4%；*vs* 减量组：77.3%；全量组：73.9%）。两试验入组患者的肾功能也有一些差距，EVE试验患者肾功能普遍正常（入组条件：CrCl>90 mL/min），而API-CAT试验（CrCl<50 mL/min比例：减量组：13.3%；全量组：14.1%），肾功能不全患者比例更加接近真实世界，其余的癌症分期、体能状态都比较接近，而两项研究均未提及肺癌患者的亚型对于出血风险的影响，综合来看，API-CAT试验的结论更适合肺癌合并PE这一人群，未来还需针对肺癌的队列对照进一步完善证据空白。

## 5 总结

肺癌合并PE患者的全程管理包括评估、诊断、治疗、预防的全过程，从评估VTE发生风险到评估是否存在PE，再到针对PE治疗，以及治疗后的二级预防，本综述遵循这一思路，梳理了目前肺癌合并PE患者的治疗和管理要点，目前大多数研究都是针对泛癌种，尤其是在抗凝药物的选择方面，针对肺癌的专属性欠缺。一方面，不同分子亚型的肺癌患者VTE发生率不同，预测肺癌患者PE的相关特异性指标研究方兴未艾；另一方面，现有的评估量表针对肺癌这一特异群体的预测能力有限，新型的生物标志物仍然缺乏大量临床数据和实验的支持，且未纳入量表用于风险评估。在进行肺癌合并VTE/PE患者治疗决策时，需要遵循指南的推荐，若无胃、食管病变的患者，首选DOACs治疗，疗程上≥3个月，期间定期评估抗凝的获益与风险。而对于有特殊疾病的患者，推荐予LMWH治疗，并且还要遵循患者的个人偏好进行综合考虑。

展望未来，针对肺癌合并PE患者，需构建高效安全的防治策略和管理路径。着重以风险评估到治疗方案的个体化，建立基于生物标志物和基因检测的精准治疗策略，关注药物间的相互作用及二级预防方案的优化。同时，致力于纳入新型的生物标志物，构建机器模型，推动诊疗模式向数智化转型，以期进一步改善患者的生存时间和生活质量。
